# Reduction of Hepatitis B Surface Antigen May Be More Significant in PEGylated Interferon-Alpha Therapy Combined with Nucleotide Analogues than Combined with Nucleoside Analogues in Chronic Hepatitis B Patients: A Propensity Score Matching Study

**DOI:** 10.1155/2022/4325352

**Published:** 2022-12-07

**Authors:** Yiran Xie, Haoxiang Zhu, Yifei Guo, Zhenxuan Ma, Xun Qi, Feifei Yang, Richeng Mao, Jiming Zhang

**Affiliations:** ^1^Department of Infectious Diseases, Shanghai Key Laboratory of Infectious Diseases and Biosafety Emergency Response, Shanghai Institute of Infectious Diseases and Biosecurity, National Medical Center for Infectious Diseases, Huashan Hospital, Fudan University, Shanghai, China; ^2^Shanghai Public Health Center of Fudan University, Shanghai, China; ^3^Department of Infectious Diseases, Jing'An Branch of Huashan Hospital, Fudan University, Shanghai, China

## Abstract

**Background:**

Nucleotide analogues (NTs) monotherapy may have a more significant effect on reducing hepatitis B surface antigen (HBsAg) than nucleoside analogues (NSs) due to their immunomodulatory function. However, this superiority remains unknown when combined with PEGylated interferon *α* (PegIFN*α*). Therefore, this study aimed to explore whether NTs have more significant antiviral effects than NSs in combination therapy with PegIFN*α*.

**Methods:**

Chronic hepatitis B (CHB) patients treated with PegIFN*α* plus nucleos(t)ide analogues (NAs) were retrospectively recruited. Efficacy and the predictors of hepatitis B surface antigen (HBsAg) reduction >1 log_10_ IU/mL after 48 weeks were analyzed.

**Results:**

A total of 95 patients were included and divided into the PegIFN*α* + NTs group and the PegIFN*α* + NSs group. Propensity score matching (PSM) was performed. The PegIFN*α* + NTs group had a greater reduction of HBsAg (−3.52 vs. −2.33 log_10_ IU/mL, *P*=0.032) and a higher proportion of patients with HBsAg reduction >1 log_10_ IU/mL (100.0% vs. 72.2%, *P*=0.003) even after PSM. However, HBsAg and hepatitis B e-antigen (HBeAg) loss rates, HBeAg seroconversion rates, degree of HBeAg and hepatitis B virus (HBV) DNA decline, HBV DNA undetectable rates, and alanine aminotransferase (ALT) normalization rates showed no significant differences. Subgroup analyses showed the difference in the reduction of HBsAg was particularly evident in HBeAg-positive and the “add-on” subgroups. PegIFN*α* plus NTs (OR = 36.667, 95% CI = 3.837–350.384) was an independent predictor for HBsAg reduction >1 log_10_ IU/mL after 48 weeks.

**Conclusion:**

This study suggests that PegIFN*α* plus NTs may lead to more HBsAg reduction, especially in HBeAg-positive and “add-on” patients.

## 1. Introduction

Chronic hepatitis B (CHB) is a global infectious disease. There are currently about 70 million people infected with hepatitis B virus (HBV) in China, with more than 20 million CHB patients. These patients are at high risks of liver cirrhosis and hepatocellular carcinoma (HCC), especially in developing countries [1], presenting an immense medical burden [2]. Covalently closed circular DNA (cccDNA) persistence within hepatocytes is relevant for chronic HBV infection [3]. Hepatitis B surface antigen (HBsAg) is a surrogate marker for cccDNA transcriptional activity [3–5]. The disappearance of HBsAg, accompanied by a sustained virological response, loss of hepatitis B e-antigen (HBeAg), recovery of alanine aminotransferase (ALT), and improvement of liver tissue lesions, is defined as functional cure. Thus, important guidelines consider sustained HBsAg disappearance after drug withdrawal as an ideal treatment endpoint [6, 7].

However, HBsAg loss is not common with current standard antiviral strategies, including nucleos(t)ide analogues (NAs) and PEGylated interferon-alpha (PegIFN*α*). Reduced HBsAg level is often associated with better outcomes, including minimizing cirrhosis and HCC, and is conducive to HBsAg clearance. Therefore, it is often used as an efficacy indicator. NAs are economical and convenient but cannot directly act on cccDNA. Patients usually need to take long-term or even life-long medications, bringing unavoidable economic and psychological burdens and drug resistance problems. In contrast, PegIFN*α* can reduce HBsAg more thoroughly in a subset of patients [8]. The low virologic response rate in PegIFN*α* monotherapy and poor reduction of HBsAg in NAs monotherapy shed some light on combination strategies.

Previous studies have proven that PegIFN*α* combined with NAs had better clinical effects than those of PegIFN*α* or NAs monotherapy [9–11], particularly in reducing HBsAg [12] and enhancing HBsAg loss rate [13]. Additionally, NAs can vary in efficacy. Nucleotide analogues (NTs), including tenofovir disoproxil fumarate (TDF), adefovir dipivoxil (ADV), and tenofovir alafenamide (TAF), are not only structurally but also functionally different from nucleoside analogues (NSs) like entecavir (ETV) and lamivudine (LAM). According to a small randomized controlled trial, the reduction in HBsAg was significantly higher in the TDF arm than in the ETV arm in NAs-naive patients [14]. Furthermore, switching from ETV to TDF or TAF significantly declines HBsAg [15, 16]. Interestingly, NTs have also been found with an additional immunological effect in interferon lambda 3 (IFN*λ*3) induction compared to NSs [17]. Meanwhile, in some studies, TDF treatment was associated with a significantly lower risk of HCC than ETV [18, 19]. Still, the comparison remains controversial [20]. The HBsAg clearance rate could reach 9.1% after 48 weeks of therapy combining PegIFN*α* and TDF followed by TDF monotherapy until 72 weeks [9]. But the rate was only 0.8% when PegIFN*α* was combined with ETV for 48 weeks and followed up to even 96 weeks [11]. According to this indirect comparison, PegIFN*α* combined with TDF (which represents NTs) appears to reach a better HBsAg clearance rate than that of PegIFN*α* combined with ETV (which represents NSs) when the treatment durations are similar. However, the populations and the end-points were not totally consistent between the two studies, making comparison difficult. There is currently no study directly comparing the efficacy of these two combination therapies.

Therefore, comparing HBsAg reduction efficacy for PegIFN*α* therapy combined with NTs or NSs in CHB patients is valuable. Thus, we conducted a retrospective study using the data of CHB patients treated with a combination of PegIFN*α* plus different NAs.

## 2. Methods

### 2.1. Patients

Between October 2011 and December 2018, a total of 159 consecutive PegIFN*α*-naive CHB patients who received PegIFN*α* for at least 48 weeks and combined with NAs during the course were retrospectively enrolled from two clinical centers, Huashan Hospital of Fudan University (Shanghai, China) and Shanghai Public Health Center of Fudan University (Shanghai, China). Chronic HBV infection was defined as HBsAg-positive and/or HBV DNA-positive for at least six months before enrollment. The combination therapy strategies could be “add-on” (adding NAs on during the therapy of PegIFN*α*) and “NAs-experienced” (adding PegIFN*α* to NAs treatment which had been more than one year). The NAs used back then were kept consistent with the previous type. In total, 64 patients were excluded, with four having underlying chronic hepatitis C, autoimmune hepatitis, HIV, or tumor, seven having used PegIFN*α* for more than 48 weeks when NAs were added to the therapeutic regimen, one combining nucleoside analogues with nucleotide analogues, six using the combination therapy for less than 12 weeks, and 46 having a PegIFN*α* therapy duration less than 48 weeks or incomplete data at an important timepoint. In this study, 95 patients were ultimately included, with one group including those who received PegIFN*α* combined with nucleoside analogues (ETV) (*n* = 18), and the other group including patients treated with PegIFN*α* combined with nucleotide analogues (TDF or ADV) (*n* = 77). This retrospective study was conducted under the approval of the Ethics Committee for Huashan Hospital of Fudan University and following the Declaration of Helsinki. Written informed consent was obtained for all patients included.

### 2.2. Clinical Data

All patients' baseline clinical data and laboratory test results were recorded. Clinical data included demographic data, history of chronic hepatitis B, and treatment history (name, dose, time, and medication complications). Laboratory test results included blood routine, liver function, and hepatitis B-related indicators (HBsAg tilters, HBeAg titers, and HBV DNA levels). The baseline was defined as the start of PegIFN*α* therapy. The duration of PegIFN*α* therapy was at least 48 weeks, with combination therapy for a minimum of 12 weeks. Laboratory examination results at 0, 12, 24, 36, and 48 weeks and the medication changes during the treatment (complications, dose changes, and addition or withdrawal of NAs) were recorded in detail.

### 2.3. Definitions of Treatment Response

The primary endpoint was the reduction levels of HBsAg from the baseline at 48 weeks of treatment.

Serological responses after 48 weeks: (1) proportion of patients with HBsAg reduction >1 log_10_ IU/mL from baseline; (2) HBsAg loss rate; (3) reduction levels of HBeAg from baseline at 48 weeks; (4) HBeAg loss rate and HBeAg seroconversion (HBeAg loss with the appearance of anti-HBe) rate. Virological responses after 48 weeks: (1) reduction of HBV DNA levels from baseline after 48 weeks; (2) HBV DNA undetectable rate (proportion of patients with DNA <500 IU/mL after 48 weeks according to the accuracy of the instrument at the time); (3) proportion of patients with HBsAg reduction >1 log_10_ IU/mL from baseline, and HBV DNA were undetectable after 48 weeks. Biochemical response at 48 weeks was defined as ALT normalization rate (proportion of patients with baseline ALT >1 upper limit of normal (ULN) and normal ALT after 48 weeks, ULN = 40 U/L).

### 2.4. Laboratory Measurements

Serum HBsAg levels were determined by Elecsys HBsAg II assay (Roche Diagnostics GmbH, Mannheim, Germany; linear range, 0.05–52,000 IU/mL).

HBsAg loss was defined as HBsAg <0.05 IU/mL. HBV DNA was measured using TaqMan fluorescence quantification, and the lower detection limit was 500 IU/mL. Routine biochemical and hematological tests were performed locally. The normal upper limit of ALT was 40 IU/L. Data from laboratory assessments were collected at baseline and after 12, 24, 36, and 48 weeks of treatment.

### 2.5. Statistical Analysis

HBsAg and HBV DNA levels and reduction levels were log (base 10) transformed.

The Kolmogorov–Smirnov test was conducted for normality testing. Continuous variables are represented by the mean ± standard deviation (SD) and median (interquartile range (IQR)). Independent *t*-tests were used to compare continuous variables with normally distributed data (*Z*-score between ±1.96, calculated by skewness and kurtosis), while Mann–Whitney *U* tests were used to compare continuous variables with a skewed distribution. The chi-squared test presented the categorical data as *n* (%). Differences among groups were evaluated using one-way analysis of variance (ANOVA) if the variances were homogeneous, and the LSD-T test was used for intergroup comparison. Otherwise, the Kruskal–Wallis test (K-W test) for nonparametric statistics was conducted. Multivariate logistic regression analysis was applied to determine the predictors that affected HBsAg reduction >1 log_10_ IU/mL from baseline at 48 weeks of treatment. To adjust for potential bias that could influence the results, including sample size with excessive deviation, we applied a balanced study based on the propensity score matching (PSM) technique at a 1 : 1 ratio with a caliper of 0.2 separately between the PegIFN*α* + ETV group and the PegIFN*α* + ADV group or the PegIFN*α* + ETV group and the PegIFN*α* + TDF group. Age, HBsAg, and prior treatment duration of NAs combined with PegIFN*α* were imputed for PSM. The balance of the variables between the groups was considered acceptable when the absolute value of the standard difference was less than 10%. Differences were considered significant at a two-tailed *P* < 0.05. All statistical analyses were carried out using SPSS software version 24.0 (IBM, Armonk, NY, USA).

## 3. Results

### 3.1. Baseline Characteristics

**Figure 1:**
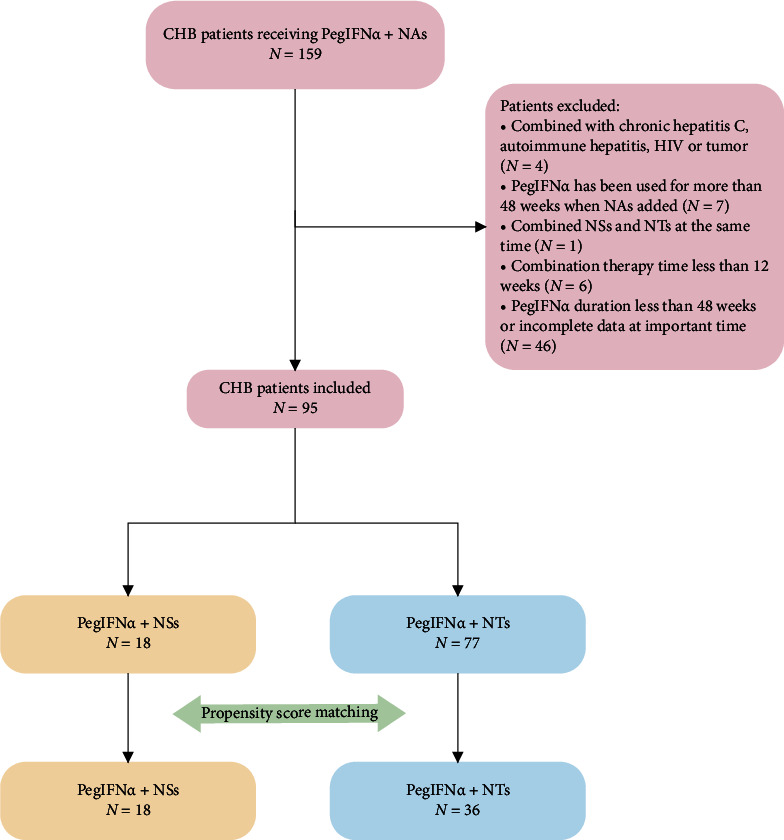
Flow diagram describing the selection of the study population.

A total of 95 cases were selected for effective analysis, including 18 patients who received a therapy combining PegIFN*α* with nucleoside analogues (PegIFN*α* + NSs) and 77 patients who received PegIFN*α* combined with nucleotide analogues (PegIFN*α* + NTs) (Figure 1). In detail, the PegIFN*α* + NTs group included the PegIFN*α* + ETV group, the PegIFN*α* + NTs group included the PegIFN*α* + ADV group and the PegIFN*α* + TDF group. There were no significant differences in baseline information between the two groups or among subgroups prior to PSM (Table 1). PSM was performed, yielding 18 patients matched in each subgroup. After PSM, relative multivariate imbalance L1 was lower than before matching, indicating a better balance. No covariate exhibited a significant imbalance, and all the covariates reached a balance within 10%, so the balance of the variables between groups was considered acceptable after PSM. No statistically significant differences were found among patients in each group after PSM (Table 1).

### 3.2. Primary Endpoint before and after PSM

**Figure 2:**
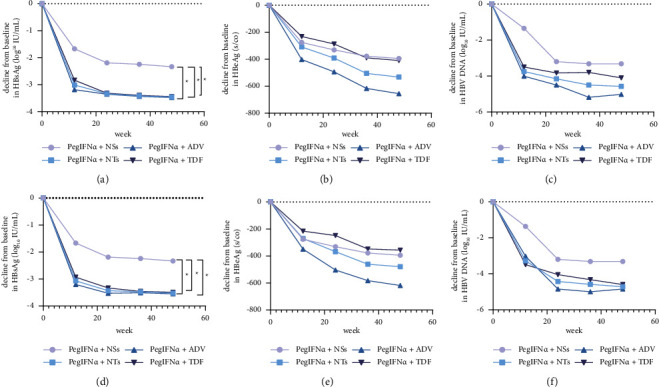
Mean reductions from baseline in different indicators. (a) HBsAg decline before matching; (b) HBeAg decline before matching; (c) HBV DNA decline before matching; (d) HBsAg decline after matching; (e) HBeAg decline after matching; (f) HBV DNA decline after matching. ^*∗*^*P* < 0.05.

HBsAg level gradually decreased during treatment. After 48 weeks, patients in the PegIFN*α* + NTs group achieved more reduction in HBsAg levels (−3.45 vs. −2.33 log_10_ IU/mL, *P*=0.040) than those in the PegIFN*α* + NSs group (Table 2). Both the PegIFN*α* + ADV group (−3.47 vs. −2.33 log_10_ IU/mL, *P*=0.029) and the PegIFN*α* + TDF group (−3.44 vs. −2.33 log_10_ IU/mL, *P*=0.046) reduced significantly more HBsAg levels than the PegIFN*α* + ETV group. After PSM, the change in HBsAg from baseline was −3.52 log_10_ IU/mL in the PegIFN*α* + NTs group and −2.33 log_10_ IU/mL (*P*=0.032) in the PegIFN*α* + NSs group (Table 3). HBsAg declined significantly more in the PegIFN*α* + NTs group (Figures 2(a) and 2(d)). Both the PegIFN*α* + ADV group (−3.55 vs. −2.33 log_10_ IU/mL, *P*=0.035) and the PegIFN*α* + TDF group (−3.49 vs. −2.33 log_10_ IU/mL, *P*=0.039) reduced HBsAg more than the PegIFN*α* + ETV group (Table 3).

### 3.3. Serological Responses

**Figure 3:**
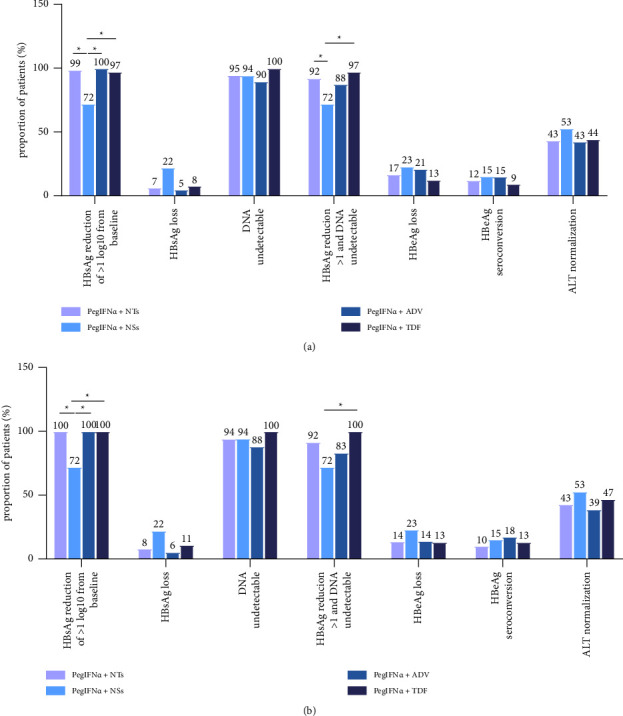
The rate of different indicators at the end of therapy. (a) Efficacy index before propensity score matching; (b) efficacy index after propensity score matching. *P* < 0.05.

Before matching, the proportion of patients with an HBsAg reduction >1 log_10_ IU/mL after 48 weeks of treatment was significantly higher in the PegIFN*α* + NTs group than in the PegIFN*α* + NSs group (98.7% vs. 72.2%, *P*=0.001). This difference was still present after matching (100% vs. 72.2%, *P*=0.003) (Figure 3). Similarly, both the PegIFN*α* + ADV group and the PegIFN*α* + TDF group had a higher proportion of HBsAg reduction >1 log_10_ IU/mL after 48 weeks than the PegIFN*α* + ETV group before and after PSM (Tables 2 and 3) (Figure 3).

We further analyzed patients who had HBsAg loss after undergoing various treatments. Before PSM, four patients (22.2%) achieved HBsAg loss in the PegIFN*α* + NSs group, while only five patients (6.5%) in the PegIFN*α* + NTs group achieved HBsAg loss, but the difference was not statistically significant (*P*=0.109) (Table 3). After PSM, patients achieving HBsAg loss in the PegIFN*α* + NTs and the PegIFN*α* + NSs group were three (8.3%) and four (22.2%), respectively, without significant statistical difference (*P*=0.205) (Figure 3). Subgroup analysis did not show statistically significant differences (Tables 2 and 3).

After 48 weeks, the reduction in serum HBeAg from baseline was more pronounced in the PegIFN*α* + NTs group than in the PegIFN*α* + NSs group both before and after PSM. However, the differences were not statistically significant (before PSM: −532.27 vs. – 394.33 s/co, *P*=0.447; after PSM: −478.72 vs. −394.33 s/co, *P*=0.667) (Tables 2 and 3) (Figures 2(b) and 2(e)). HBeAg loss at 48 weeks occurred in 11 patients (16.9%) in the PegIFN*α* + NTs group and in three patients (23.1%) in the PegIFN*α* + NSs group before matching (*P*=0.895) (Table 2). Meanwhile, eight (12.3%) and two (15.4%) patients from each group achieved HBeAg seroconversion (*P*=1.000) (Figure 3). After PSM, the HBeAg loss rate (23.1% vs. 13.8%, *P*=0.657) and HBeAg seroconversion rate (15.4% vs. 10.3.%, *P*=0.637) showed no significant differences between the two groups (Figure 3). No differences were observed among subgroups (Tables 2 and 3).

### 3.4. Virological Responses

Before matching, HBV DNA decreased by −4.57 log_10_ IU/mL from baseline in the PegIFN*α* + NTs group and −3.32 log_10_ IU/mL in the PegIFN*α* + NSs group (*P*=0.198) (Table 2). After matching, the changes in HBV DNA from baseline were −4.72 log_10_ IU/mL and −3.32 log_10_ IU/mL in patients treated with PegIFN*α* + NTs and PegIFN*α* + NSs, respectively (*P*=0.194) (Figures 2(c) and 2(f)). Meanwhile, the number of patients who reached HBV DNA below the lower detection limit (<500 IU/mL) after 48 weeks was 72 (94.7%) in the PegIFN*α* + NTs group and 17 (94.4%) in the PegIFN*α* + NSs group (*P*=1.000) before matching, and 33 (94.3%) vs. 17 (94.4%), respectively, after matching (*P*=1.000) (Figure 3). No differences were observed among subgroups (Tables 2 and 3).

Interestingly, the proportion of patients who simultaneously achieved both HBsAg reduction >1 log_10_ IU/mL and undetectable HBV DNA was 92.2% in the PegIFN*α* + NTs group and 72.2% in the PegIFN*α* + NSs group, with significant difference before matching (*P*=0.048) (Figure 3). The PegIFN*α* + TDF group had a significantly much higher rate than that of the PegIFN*α* + ETV group (97.3% vs. 72.2%, *P*=0.012). However, the proportion in the PegIFN*α* + NTs group after PSM was not significantly higher (91.7% vs. 72.2%, *P*=0.205) compared with the group treated with PegIFN*α* + NSs, still the PegIFN*α* + TDF group had a significantly higher proportion than the PegIFN*α* + ETV group (100.0% vs. 72.2%, *P*=0.045) (Table 3) (Figure 3).

### 3.5. Biochemical Responses

The proportion of patients with elevated baseline ALT who returned to normal levels at 48 weeks differed between the two groups. However, the difference was not statistically significant. In all, 33 patients (43.4%) in the PegIFN*α* + NTs group and nine patients (52.9%) in the PegIFN*α* + NSs group achieved a biochemical response of serum ALT level <40 IU/L at the end of therapy before PSM (*P*=0.476) (Table 2). After matching, 15 patients (42.9%) in the PegIFN*α* + NTs and nine patients (52.9%) in the PegIFN*α* + NSs group had biochemical responses, respectively (*P*=0.494) (Figure 3). Biochemical responses did not vary substantially by subgroups (Tables 2 and 3).

### 3.6. Subgroup Analyses

Patients were divided into subgroups based on HBeAg status or combination strategies. No significant differences were found at baseline among patients treated with PegIFN*α* plus different oral drugs in HBeAg-positive, HBeAg-negative, or “add-on” patients (Tables S1–S3). Patients treated with PegIFN*α* + NSs had a lower baseline HBV DNA level in the “NAs-experienced” subgroup (Table S4). We found that there were more “add-on” patients in the HBeAg-positive subgroup (66.7% vs. 17.6%, *P*=0.001), and the ALT level was also higher (104 vs. 34 U/L, *P*=0.001) than in the HBeAg-negative subgroup (Table S5). Patients in the “NAs-experienced” subgroup had a longer duration of antiviral therapy before adding on PegIFN*α*. Therefore, the levels of HBsAg, HBeAg, and HBV DNA were lower than the “add-on” subgroup at baseline (Table S6).

In the HBeAg-positive subgroup, the reduction of HBsAg (−3.62 vs. −2.43 log_10_ IU/mL, *P*=0.002) was significantly more and the proportion of patients with HBsAg reduction >1 log_10_ IU/mL after 48 weeks was significantly higher (100.0% vs. 69.2%, *P*=0.001) in the PegIFN*α* + NTs group (Table S7). Antiviral effects in HBeAg-negative patients seemed to have no significant differences between the PegIFN*α* + NTs group and the PegIFN*α* + NSs group, although the sample size was too small for meaningful statistical analysis (Table S8). In the “add-on” subgroup, the reduction of HBsAg was significant more in the PegIFN*α* + NTs group than in the PegIFN*α* + NSs group (−3.89 vs. −2.27 log_10_ IU/mL, *P*=0.002). HBsAg reduction was significantly more both in the PegIFN*α* + TDF group (−3.85 vs. −2.27 log_10_ IU/mL, *P*=0.008) and in the PegIFN*α* + ADV group (−3.91 vs. −2.27 log_10_ IU/mL, *P*=0.003) than in the PegIFN*α* + ETV group. The proportion of patients who achieved HBsAg reduction >1 log_10_ IU/mL was higher in the PegIFN*α* + NTs group than in the PegIFN*α* + NSs group (100.0% vs. 75.0%, *P*=0.019) (Table S9). In the “NAs-experienced” subgroup, no significant differences in the reduction of the HBsAg after 48 weeks were observed between the PegIFN*α* + NTs group and the PegIFN*α* + NSs group. However, the proportion of patients with an HBsAg reduction >1 log_10_ IU/mL at 48 weeks of treatment was significantly higher in the PegIFN*α* + NTs group than in the PegIFN*α* + NSs group (96.7% vs. 70.0%, *P*=0.042), suggesting a significant difference in antiviral efficacy (Table S10). Because all the patients in the PegIFN*α* + NSs group had undetectable viral loads at baseline, so the HBV DNA level did not drop with treatment. Therefore, HBV DNA reduction levels were not comparable between two groups. Subgroup analyses were performed using data before PSM, given our sample size.

### 3.7. Predictors Associated with HBsAg Reduction >1 log_10_ IU/mL at 48 Weeks

All patients were divided into two groups according to whether or not they achieved HBsAg reduction >1 log_10_ IU/mL after 48 weeks. Univariate analysis was performed to analyze the effect of clinical data and laboratory tests. Factors with a *P* value <0.1 or clinical significance were included in multivariate logistic regression analysis (forward: conditional method). As a result, we found that treatment with PegIFN*α* plus NTs (OR = 36.667, 95% CI = 33.837–350.384) (Table 4) was an independent predictor contributing to HBsAg reduction >1 log_10_ IU/mL at 48 weeks.

## 4. Discussion

To date, PegIFN*α* and NAs are important clinical first-line anti-HBV drugs with different mechanisms and effects on innate and adaptive immunity. NAs are oral direct antiviral drugs that reduce the viral load by inhibiting HBV DNA polymerase and reverse transcriptase. At the same time, they cannot directly inhibit the transcriptional activity of cccDNA. Therefore, obtaining durable immunological control is difficult, and the clearance and seroconversion of HBsAg and HBeAg are not easily achievable. As a result, long-term medication is often required. PegIFN*α* can enhance innate immunity, trigger T cell-mediated immune responses, prevent HBV protein formation, and deplete the cccDNA pool [21], resulting in superior effectiveness to NAs in reducing HBsAg [8]. Nearly one-third of PegIFN*α* responders achieve HBsAg clearance. In addition, strong inhibition of viral replication by NAs can assist PegIFN*α*'s immunomodulatory effect [22]. Hence, a combination strategy with PegIFN*α* plus NAs is theoretically feasible and an inevitable trend for future development. However, before a new generation of effective drugs is introduced and popularized, exploration of the combination strategy has become a major focus of current research.

There have been several studies on the efficacy of combination therapy, among which many have shown combination therapy to be superior to monotherapy in reducing HBsAg levels [9, 23, 24] and found that combination therapy could even significantly increase HBsAg loss rate (9.1% vs. 2.8%) [24]. Furthermore, compared with NAs monotherapy, combination therapy resulted in a higher percentage of HBeAg loss (26% vs. 13%, at 96 weeks) [21] and a higher HBeAg seroconversion rate (15% vs. 5%, at 48 weeks) [25] as well. Therefore, it is evident that combination therapy has prominent advantages over monotherapy. However, combination therapy's baseline conditions, optimal treatment duration, and sustained response rate require further exploration.

At the same time, it is unclear whether efficacy differs between nucleotide analogues and nucleoside analogues when combined with PegIFN*α*. The two oral drugs are functionally different, especially in HBsAg reduction. Koike et al. found that TDF reduced significantly more HBsAg levels at week 24 (–0.147 vs. –0.027 log_10_ IU/mL, *P* < 0.05) and 48 (–0.208 vs. –0.051 log_10_ IU/mL, *P* < 0.05) in NAs-naive patients [14]. Furthermore, HBeAg-negative patients whose HBsAg had not been reduced during a 48-week ETV treatment had a significantly higher HBsAg reduction after switching to TDF or TAF than in the ETV continuation group [15]. HBV infection is a risk factor for hepatocarcinogenesis. Nevertheless controversial, previous research has shown that TDF treatment could be associated with a lower risk of HCC than ETV treatment. A large retrospective analysis in China found that over a median follow-up time of 3.6 years, 4.9% of ETV-treated patients developed HCC, while it occurred in only 0.6% of TDF-treated patients [19]. Similarly, a study in Korea consistently found that the annual incidence rate of HCC was significantly lower in the TDF group than in the ETV group (0.64 vs. 1.06 per 100 person-year) [18]. Notably, researchers have indicated that patients treated with nucleotide analogues, especially ADV, have higher serum IFN*λ*3 levels than those treated with nucleoside analogues [26, 27]. The ability of IFN*λ*3 to induce interferon-stimulated genes (ISGs) in Huh7 cell lines is stronger than that of interferon lambda 1/2 (IFN-*λ*1/*λ*2), and this ability is weaker but longer-lasting than that of IFN*α* [26]. ISGs can encode antiviral proteins via complex intracellular signaling pathways, implying that IFN*λ*3 may be more effective against viruses than IFN*α*. Recombinant IFN*λ*3 had been shown to reduce HBsAg levels in vitro and had an additive antiviral effect with IFN*α* [17], further regulating the secretion of cytokines and enhancing antiviral immune function [28]. Hence, we supposed that a combination of PegIFN*α* with nucleotide analogues could have a better effect on reducing HBsAg levels than with nucleoside analogues. According to Ahn et al., the HBsAg clearance rate could reach 9.1% after 48 weeks of therapy combining PegIFN*α* and TDF, followed by TDF monotherapy until 72 weeks. No patient achieved HBsAg clearance in the TDF monotherapy group [9]. Liem et al. found that when PegIFN*α* was combined with ETV for 48 weeks and followed up for 96 weeks, only 0.8% of patients achieved HBsAg loss. No patients in the ETV monotherapy group achieved HBsAg clearance [11]. On the contrary, there are meta-analyses showing that the differences in HBsAg loss rates at the end of the combination therapy are not statistically significant among different NAs (ETV 11% vs. ADV 12% vs. LAM 9% vs. TDF 6%, *P* > 0.05) and have found similar results for the HBsAg seroconversion rate (5% vs. 5% vs. 9% vs. 4%, *P* > 0.05) [29]. A prospective follow-up study found HBsAg loss occurred similarly in PegIFN + ADV (18.6%) and PegIFN + TDF (11.7%) patients up to five years after the end of a 48-week combination therapy. This study, however, did not provide the result when PegIFN combined with NSs [30]. Lin et al. recently found that the addition of TDF to Peg-IFN*α*-2b in HBeAg-positive CHB patients with a poor response after 12 weeks of Peg-IFN*α*-2b monotherapy reduced HBsAg significantly more than the addition of ETV to Peg-IFN*α*-2b (−1.799 log_10_ IU/mL vs. −1.078 log_10_ IU/mL, *P*=0.0491) [31]. It was an important result as it compared the addition of TDF or ETV to Peg-IFN*α*-2b directly. However, considering the small sample size and the restrictive conditions for the selected population, it lacks universality, and a larger sample size study is required to verify the results. Therefore, whether PegIFN*α* combined with different NAs influences HBsAg reduction and clearance is still unclear. The loss rate of HBeAg after 48 weeks was similar between PegIFN*α* + TDF and PegIFN*α* + ETV (29.0% vs. 31.0%) [32]. Recent data from another study pointed out that PegIFN*α* combined with TDF could improve HBeAg responses in a short time. No advantages were found when PegIFN*α* was combined with LAM or ETV [33]. However, Lin et al. showed that the HBeAg loss rate was significantly higher in the TDF add-on group than in the ETV add-on group after 48 weeks (40% vs. 10%, *P*=0.028) [31]. Interestingly, these studies suggested that PegIFN*α* combined with different NAs could have different efficacies, but direct evidence was required, and the mechanism underlying the differences must be discussed. We conducted this retrospective study to provide this evidence based on these findings. TAF has only been launched in recent years, and with insufficient studies discussing the efficacy of PegIFN*α* plus TAF, we did not include patients who received TAF in the current study. Meanwhile, patients in our cohort who used LAM were excluded according to the exclusion criteria, so ETV was the only nucleoside analogues analyzed. To our knowledge, this study was the first to retrospectively compare HBsAg level reduction efficacy for CHB patients treated with different NAs in PegIFN*α* combination therapy, no matter which combination strategy was adopted. This could help prove that the difference in reduction was due to the types of NAs.

In order to minimize the impact of bias, PSM was performed to eliminate the inequality caused by excessive deviation of the general data and sample size. After PSM, the results showed that the HBsAg levels of the PegIFN*α* + NSs group decreased by an average of −2.33 log_10_ IU/mL from baseline at 48 weeks, while it decreased significantly more in the PegIFN*α* + NTs group, by an average of −3.52 log_10_ IU/mL (*P*=0.032). The reductions of HBsAg in both groups were more than those in Lin et al.' study [31]. This could be because our study had a longer combination course and some patients had previously received NA treatment. The proportion of patients achieving HBsAg reduction >1 log_10_ IU/mL was significantly higher at 48 weeks in the PegIFN*α* + NTs group compared to the PegIFN*α* + NSs group (100% vs. 72.2%, *P*=0.003). However, even after PSM adjustment, no significant differences in the following indicators were found between the two groups: HBsAg loss rate, HBV DNA reduction levels, HBeAg reduction levels, HBeAg loss rate, HBeAg seroconversion rate, HBV DNA undetectable rate, and ALT normalization rate. The observation endpoint of this study was the 48th week of treatment, and subsequent follow-up had not yet been carried out, resulting in difficulty achieving HBsAg clearance, especially for antiviral treatment-naive patients. The ability to maintain steady HBsAg clearance after combination therapy cannot be confirmed. Another reason for the significant differences in decline levels, but not in HBsAg loss rates, maybe the small sample size. Based on the results of our study, we believe that NTs may significantly reduce more HBsAg than NSs when combined with PegIFN*α*. This reduction will contribute to achieving HBsAg clearance and even a functional cure. In our study, the proportion of patients who simultaneously reached HBV DNA below the lower detection limit and HBsAg reduction >1 log_10_ IU/mL from baseline at 48 weeks differed between the PegIFN*α* + ETV group and the PegIFN*α* + TDF group after PSM (100.0% vs. 72.2%, *P*=0.045). This result exemplifies the dual effectiveness of PegIFN*α* combination therapy with TDF over combination therapy with ETV in inhibiting viral replication and reducing HBsAg levels simultaneously. Furthermore, the multivariate logistic regression also showed that treatment with PegIFN*α* plus NTs was an independent predictor for HBsAg decline >1 log_10_ IU/mL at 48 weeks, suggesting that the combination of PegIFN*α* and NTs can fasten HBsAg decline.

Combination strategies have been studied, including “De novo,” “NAs-experienced,” “add-on,” and “switch-to.” Several studies have shown that the “NAs-experienced” strategy seemed the best. The “switch-to” strategy was particularly effective and improved the HBsAg clearance rate [13, 29, 34]. This maybe because the direct antiviral activity of NAs can lead to virological suppression, which can further improve the immunomodulatory effect of PegIFN*α*, thereby maximizing the advantages of combination therapy. Since patients with different combination strategies and HBeAg status were enrolled, subgroup analyses were performed using data before PSM to determine whether the antiviral effects were different between “add-on” and “NAs-experienced” subgroups as well as between HBeAg-positive and HBeAg-negative subgroups. We found that in HBeAg-positive patients, the reduction of HBsAg was significantly more in the PegIFN*α* + NTs group. Possible mechanism may be the additional immunomodulatory effects of NTs combined with PegIFN*α*, which deregulate the immunosuppression caused by HBeAg [35, 36], resulting in a better clinical efficacy. However, further research is needed to investigate the different effects of NTs and NSs on the immune system. Regrettably, the number of HBeAg-negative patients was relatively small and was prone to bias. Therefore, no statistical analysis of this subpopulation was conducted, and further studies are warranted to confirm our findings. More significant reduction of HBsAg in the PegIFN*α* + NTs group was also observed in the “add-on” subgroup. Besides, patients who achieved a reduction in HBsAg >1 log_10_ IU/m were significantly more in the PegIFN*α* + NTs group in HBeAg-positive, “add-on,” and “NAs-experienced” patients. We, therefore, infer that our findings were generally consistent across subgroups.

Limitations of our study include that it is a retrospective study with small sample size and short therapy duration without a long-term follow-up. Furthermore, the combination strategy was not precisely uniform, although the combination therapy duration was guaranteed at least 24 weeks. However, the prior treatment duration and drugs before combination for NAs-experienced patients, the weeks of adding-on NAs for “add-on” patients, and the total weeks of combination at baseline before and after PSM were not statistically different. In addition, the results were partially observable in subgroup analyses. So, the following analysis was considered reliable. However, further randomized controlled trials are required for verification.

## 5. Conclusion

In conclusion, reduction of HBsAg might be more pronounced in PegIFN*α* therapy combined with NTs than NSs, especially in HBeAg-positive patients and patients using “add-on” strategies. This finding will be beneficial for promoting further HBsAg clearance and functional cure. In addition, this finding can be used to make clinical decisions. Therefore, similar findings and mechanisms should be investigated further [37].

## 6. Consent

Informed consent was obtained from all patients.

## Figures and Tables

**Table 1:** Baseline characteristics of the patients before and after PSM.

Variables	Before PSM			*P*	After PSM			*P*
	PegIFN*α* + NSs (*n* = 18)	PegIFN*α* + NTs (*n* = 77)			PegIFN*α* + NSs (*n* = 18)	PegIFN*α* + NTs (*n* = 36)		
	PegIFN*α* + ETV (*n* = 18)	PegIFN*α* + ADV (*n* = 40)	PegIFN*α* + TDF (*n* = 37)		PegIFN*α* + ETV (*n* = 18)	PegIFN*α* + ADV (*n* = 18)	PegIFN*α* + TDF (*n* = 18)	
NAs-experienced^c^	10 (55.6)	30 (39.0)		0.199	10 (55.6)	13 (36.1)		0.173
	10 (55.6)	13 (32.5)	17 (45.9)	0.215	10 (55.6)	6 (33.3)	7 (38.9)	0.374

Duration of NAs before combined with PegIFN*α* (week)^b^	96 (42–168)	48 (14–192)		0.397	96 (42–168)	96 (10–384)		0.948
	96 (42–168)	96 (24–384)	48 (11–60)	0.085	96 (42–168)	384 (170–456)	32 (9–96)	0.105

Weeks of adding-on PegIFN*α* (week)^a^	10.33 ± 13.90	10.94 ± 12.49		0.857	10.33 ± 13.90	11.67 ± 11.88		0.715
	10.33 ± 13.90	12.03 ± 11.58	9.76 ± 13.45	0.728	10.33 ± 13.90	13.33 ± 11.56	10.00 ± 12.29	0.685

Total weeks of combination (week)^a^	36.50 ± 13.86	36.60 ± 12.74		0.977	36.50 ± 13.86	36.33 ± 13.86		0.964
	36.50 ± 13.86	35.09 ± 12.02	38.24 ± 13.44	0.564	36.50 ± 13.86	34.7 ± 11.56	38.0 ± 12.29	0.731

Age (years)^a^	37.2 ± 6.3	34.8 ± 7.7		0.222	37.22 ± 6.32	34.56 ± 6.43		0.154
	37.2 ± 6.3	35.5 ± 13.9	34.1 ± 8.5	0.332	37.22 ± 6.32	34.56 ± 6.0	34 ± 6.9	0.260

Male^c^	17 (94.4)	59 (76.6)		0.169	17 (94.4)	29 (80.6)		0.343
	17 (94.4)	31 (77.5)	28 (75.7)	0.230	17 (94.4)	15 (83.3)	14 (77.8)	0.318

HBeAg-positive^c^	13 (72.2)	65 (84.4)		0.382	13 (72.2)	29 (80.6)		0.728
	13 (72.2)	33 (82.5)	32 (86.5)	0.431	13 (72.2)	14 (77.8)	15 (83.3)	0.725

BMI (kg/cm^2^)^a^	23.64 ± 2.04	22.53 ± 2.60		0.205	23.67 ± 2.04	22.38 ± 1.78		0.072
	23.64 ± 2.04	22.66 ± 2.58	22.40 ± 2.66	0.887	23.67 ± 2.04	22.3 ± 1.9	22.5 ± 1.7	0.195

WBC (×10^9^/L)^a^	5.1 ± 0.7	5.2 ± 1.3		0.259	5.8 ± 1.5	5.2 ± 1.3		0.113
	5.1 ± 0.7	5.1 ± 1.5	5.3 ± 0.9	0.245	5.8 ± 1.5	5.2 ± 1.7	5.2 ± 0.8	0.287

NEUT (%)^b^	62 (53–67)	55 (48–63)		0.076	58 (51–68)	56 (48–63)		0.246
	62 (53–67)	57 (48–63)	57 (48–63)	0.174	58 (51–68)	55 (50–63)	56 (47–64)	0.510

RBC (×10^9/L)^a^	5.8 ± 1.2	4.8 ± 0.5		0.351	5.1 ± 0.4	4.9 ± 0.2		0.144
	5.8 ± 1.2	4.9 ± 0.4	5.0 ± 0.6	0.308	5.1 ± 0.4	4.9 ± 0.1	4.9 ± 0.3	0.287

HGb (g/L)^a^	156 ± 10	152 ± 14		0.260	156 ± 10	152 ± 14		0.244
	156 ± 10	152 ± 14	153 ± 15	0.504	156 ± 10	152 ± 15	152 ± 14	0.510

PLT (×10^9/L)^a^	198 ± 47	193 ± 42		0.671	198 ± 47	191 ± 43		0.582
	198 ± 47	185 ± 44	202 ± 38	0.223	198 ± 47	182 ± 43	201 ± 41	0.392

ALB (U/L)^a^	48 ± 3	46 ± 4		0.169	48 ± 3	46 ± 4		0.091
	48 ± 3	46 ± 3	47 ± 4	0.109	48 ± 3	46 ± 4	46 ± 4	0.203

ALT (U/L)^b^	48 (32–153)	97 (34–209)		0.269	48 (32–153)	99 (34–234)		0.210
	48 (32–153)	111 (34–263)	90 (35–183)	0.340	48 (32–153)	97 (34–279)	101 (35–202)	0.407

ALT > ULN^c^	11 (61.1)	50 (69.4)		0.499	11 (61.1)	24 (70.6)		0.488
	11 (61.1)	26 (72.2)	24 (66.7)	0.700	11 (61.1)	12 (70.6)	12 (70.6)	0.786

AST (U/L)^b^	27 (23–75)	48 (23–94)		0.214	27 (23–75)	53 (25–102)		0.136
	27 (23–75)	48 (22–98)	47 (24–91)	0.415	27 (23–75)	42 (22–111)	57 (26–99)	0.314

GGT (U/L)^b^	23 (18–63)	18 (26–47)		0.980	23 (18–63)	33 (20–53)		0.610
	23 (18–63)	29 (17–57)	24 (20–35)	0.958	23 (18–63)	36 (18–58)	32 (22–45)	0.860

TBIL (*μ*mol/L)^a^	13.1 ± 7.5	13.1 ± 7.0		0.980	13.1 ± 7.5	14.1 ± 9.0		0.677
	13.1 ± 7.5	12.4 ± 4.5	13.9 ± 8.9	0.676	13.1 ± 7.5	13.2 ± 4.7	15.1 ± 12.2	0.745

HBsAg (log_10_ IU/mL)^a^	3.25 ± 1.05	3.64 ± 0.91		0.116	3.25 ± 1.05	3.71 ± 0.86		0.091
	3.25 ± 1.05	3.67 ± 0.90	3.61 ± 0.93	0.282	3.25 ± 1.05	3.77 ± 1.0	3.66 ± 0.7	0.228

HBsAg > 250 IU/mL^c^	15 (83.3)	71 (92.2)		0.477	15 (83.3)	33 (91.7)		0.388
	15 (83.3)	36 (90.0)	35 (94.6)	0.417	15 (83.3)	16 (88.9)	17 (94.4)	0.557

HBeAg (s/co)^a^	411.38 ± 517.81	510.20 ± 617.34		0.552	411.38 ± 517.81	449.20 ± 616.64		0.832
	411.38 ± 517.81	606.30 ± 95.87	406.40 ± 621.28	0.303	411.38 ± 517.81	568.45 ± 639.86	329.96 ± 585.93	0.469

HBV DNA (log_10_ IU/mL)^a^	4.68 ± 2.30	5.41 ± 2.21		0.241	4.68 ± 2.30	5.48 ± 2.20		0.249
	4.68 ± 2.30	5.78 ± 2.13	4.68 ± 2.29	0.214	4.68 ± 2.30	5.66 ± 2.18	5.33 ± 2.28	0.479

^a^Variables are expressed as mean ± SD; ^b^variables are expressed as median (IQR); ^c^variables are expressed as *n* (%). ADV, adefovir dipivoxil; ALB, albumin; ALT, alanine aminotransferase; AST, aspartate transaminase; BMI, body mass index; ETV, entecavir; GGT, gamma glutamyl transferase; HBeAg, hepatitis B e antigen; HBsAg, hepatitis B surface antigen; HGb, hemoglobin; IQR, interquartile range; NAs, nucleos(t)ide analogues; NEUT, neutrophils; NSs, nucleoside analogues; NTs, nucleotide analogues; PegIFN*α*, PEGylated interferon *α*; PLT, platelet; RBC, red blood cells; SD, standard deviation; TBIL, total bilirubin; TDF, tenofovir disoproxil fumarate; ULN, upper limit of normal; WBC, white blood cells.

**Table 2:** Efficacy results at week 48 before PSM.

Responses	PegIFN*α* + NSs (*n* = 18)	PegIFN*α* + NTs (*n* = 77)		*P* (total)	*P* (ETV vs. ADV)	*P* (ETV vs. TDF)	*P* (ADV vs. TDF)
	PegIFN*α* + ETV (*n* = 18)	PegIFN*α* + ADV (*n* = 40)	PegIFN*α* + TDF (*n* = 37)				
HBsAg reduction from baseline at week 48, log_10_ IU/mL	–2.33	–3.45		0.040			
	–2.33	–3.47	–3.44	0.082	0.029	0.046	0.901

HBeAg reduction from baseline at week 48, s/co	–394.33	–532.37		0.447			
	–394.33	–654.90	–409.83	0.167	0.175	0.937	0.091

HBV DNA reduction from baseline at week 48, log_10_ IU/mL	–3.32	–4.57		0.198			
	–3.32	–5.02	–4.10	0.251	0.112	0.481	0.287

HBsAg loss, *n* (%)	4 (22.2)	5 (6.5)		0.109			
	4 (22.2)	2 (5.0)	3 (8.1)	0.152	0.068	0.200	0.667

HBeAg loss, *n* (%)	3 (23.1)	11 (16.9)		0.895			
	3 (23.1)	7(21.2)	4 (12.5)	0.562	1.000	0.394	0.349

HBeAg seroconversion, *n* (%)	2 (15.4)	8 (12.3)		1.000			
	2 (15.4)	5 (15.2)	3 (9.4)	0.742	1.000	0.617	0.708

HBV DNA undetectable, *n* (%)	17 (94.4)	72 (94.7)		1.000			
	17 (94.4)	35 (89.7)	37 (100)	0.062	1.000	0.327	0.116

HBsAg reduction >1 log_10_ from baseline, *n* (%)	13 (72.2)	76 (98.7)		0.001			
	13 (72.2)	40 (100)	36 (97.3)	0.004	0.026	0.035	0.481

HBsAg reduction >1 log_10_ and DNA undetectable, *n* (%)	13 (72.2)	71 (92.2)		0.048			
	13 (72.2)	35 (87.5)	36 (97.3)	0.024	0.258	0.012	0.202

ALT normalization, *n* (%)	9 (52.9)	33 (43.4)		0.476			
	9 (52.9)	17 (42.5)	16 (44.4)	0.764	0.469	0.563	0.864

ADV, adefovir dipivoxil; ALT, alanine aminotransferase; ETV, entecavir; HBeAg, hepatitis B e-antigen; HBsAg, hepatitis B surface antigen; HBV, hepatitis B virus; NSs, nucleoside analogues; NTs, nucleotide analogues; PegIFN*α*, PEGylated interferon *α*; TDF, tenofovir disoproxil fumarate.

**Table 3:** Efficacy results at week 48 after PSM.

Responses	PegIFN*α* + NSs (*n* = 18)	PegIFN*α* + NTs (*n* = 36)		*P* (total)	*P* (ETV vs. ADV)	*P* (ETV vs. TDF)	*P* (ADV vs. TDF)
	PegIFN*α* + ETV (*n* = 18)	PegIFN*α* + ADV (*n* = 18)	PegIFN*α* + TDF (*n* = 18)				
HBsAg reduction from baseline at week 48, log_10_ IU/mL	–2.33	–3.52		0.032			
	–2.33	–3.55	–3.49	0.092	0.035	0.039	0.853

HBeAg reduction from baseline at week 48, s/co	–394.33	–478.72		0.667			
	–394.33	–618.26	–356.63	0.417	0.301	0.862	0.236

HBV DNA reduction from baseline at week 48, log_10_ IU/mL	–3.32	–4.72		0.194			
	–3.32	–4.85	–4.60	0.426	0.240	0.311	0.840

HBsAg loss, *n* (%)	4 (22.2)	3 (8.3)		0.205			
	4 (22.2)	1 (5.6)	2 (11.1)	0.316	0.338	0.658	1.000

HBeAg loss, *n* (%)	3 (23.1)	4 (13.8)		0.657			
	3 (23.1)	2 (14.3)	2 (13.3)	0.764	0.648	0.639	1.000

HBeAg seroconversion, *n* (%)	2 (15.4)	3 (10.3)		0.637			
	2 (15.4)	1 (7.1)	2 (13.3)	0.773	0.596	1.000	1.000

HBV DNA undetectable, *n* (%)	17 (94.4)	33 (94.3)		1.000			
	17 (94.4)	15 (88.2)	18 (100.0)	0.221	0.603	1.000	0.229

HBsAg reduction >1 log_10_ from baseline, *n* (%)	13 (72.2)	36 (100.0)		0.003			
	13 (72.2)	18 (100.0)	18 (100.0)	0.002	0.045	0.045	—

HBsAg reduction >1 log_10_ and DNA undetectable, *n* (%)	13 (72.2)	33 (91.7)		0.205			
	13 (72.2)	15 (83.3)	18 (100.0)	0.042	0.691	0.045	0.229

ALT normalization, *n* (%)	9 (52.9)	15 (42.9)		0.494			
	9 (52.9)	7 (38.9)	8 (47.1)	0.704	0.404	0.732	0.625

ADV, adefovir dipivoxil; ALT, alanine aminotransferase; ETV, entecavir; HBeAg, hepatitis B e-antigen; HBsAg, hepatitis B surface antigen; HBV, hepatitis B virus; NSs, nucleoside analogues; NTs, nucleotide analogues; PegIFN*α*, PEGylated interferon *α*; TDF, tenofovir disoproxil fumarate.

**Table 4:** Multivariate logistic regression of HBsAg reduction >1 log_10_ IU/mL at 48 weeks.

Predictors	Univariate analysis		Multivariate analysis	
	OR (95% CI)	*P*	OR (95% CI)	*P*
Age (years)	0.858 (0.780–0.944)	0.002		
Age > 40 years	22.812 (2.492–208.817)	0.006		
BMI (kg/cm^2^)	0.753 (0.479–1.183)	0.218		
HBeAg-positive	0.405 (0.068–2.418)	0.322		
NAs-experienced	0.340 (0.059–1.953)	0.226		
PegIFN*α* plus NTs	29.231 (3.155–270.801)	0.003	36.667 (3.837–350.384)	0.002
PegIFN*α* plus NSs	1.181 (0.129–10.774)	0.883		
Week of PegIFN*α* adding NAs (week)	0.992 (0.931–1.058)	0.813		
Weeks of NAs before adding PegIFN*α* (week)	1.003 (0.992–1.015)	0.565		
Total weeks of combination (week)	1.004 (0.942–1.070)	0.909		
HBeAg at baseline (s/co)	1.001 (0.999–1.004)	0.263		
ALT at baseline (U/L)	1.007 (0.995–1.018)	0.259		
ALT > ULN	4.720 (0.812–27.452)	0.084		
ALT at week 12 (U/L)	1.025 (0.993–1.058)	0.124		
HBsAg at baseline (IU/mL)	3.338 (1.479–7.533)	0.004		
HBsAg > 250 IU/mLat baseline	5.857 (0.908–37.798)	0.063		
HBsAg at week 12 (IU/mL)	1.000 (1.000–1.001)	0.362		
HBsAg at week 24 (IU/mL)	1.000 (1.000–1.001)	0.310		
HBsAg decline at week 12 (log_10_ IU/mL)	0.813 (0.507–1.303)	0.390		
HBsAg decline at week 24 (log_10_ IU/mL)	0.538 (0.310–0.932)	0.027		
HBV DNA at baseline (log_10_ IU/mL)	1.317 (0.837–2.074)	0.234		
HBV DNA at week 12 (log_10_ IU/mL)	1.000 (1.000–1.000)	0.543		
HBV DNA decline at week 12 (log_10_ IU/mL)	0.905 (0.749–1.093)	0.300		
WBC (×10^9^/L)	0.910 (0.451–1.836)	0.792		
NEUT (%)	0.962 (0.874–1.058)	0.420		
RBC (×10^9^/L)	1.959 (0.394–9.734)	0.411		
HGb (g/L)	0.966 (0.898–1.039)	0.350		
PLT (×10^9^/L)	1.024 (1.001–1.048)	0.037		0.040
ALB (U/L)	1.055 (0.793–1.404)	0.711		
AST (U/L)	1.018 (0.985–1.051)	0.293		
GGT (U/L)	1.023 (0.963–1.086)	0.461		
TBIL (*μ*mol/L)	1.020 (0.888–1.172)	0.779		

ADV, adefovir dipivoxil; ALB, albumin; ALT, alanine aminotransferase; AST, aspartate transaminase; BMI, body mass index; ETV, entecavir; GGT, gamma glutamyl transferase; HBeAg, hepatitis B e-antigen; HBsAg, hepatitis B surface antigen; HGb, hemoglobin; NEUT, neutrophils; NAs, nucleos(t)ide analogues; NEUT, neutrophils; NSs, nucleoside analogues; NTs, nucleotide analogues; PegIFN*α*, PEGylated interferon *α*; PLT, platelet; RBC, red blood cells; TBIL, total bilirubin; TDF, tenofovir disoproxil fumarate; ULN, upper limit of normal; WBC, white blood cells.

## Data Availability

All data generated or analyzed during this study are included within the article.

## Abbreviations

ADV: Adefovir dipivoxil
ALB: Albumin
ALT: Alanine aminotransferase
AST: Aspartate transaminase
BMI: Body mass index
cccDNA: Covalently closed circular DNA
CI: Confidence interval
CHB: Chronic hepatitis B
ETV: Entecavir
GGT: Gamma glutamyl transferase
HBeAg: Hepatitis B e-antigen
HBsAg: Hepatitis B surface antigen
HBV: Hepatitis B virus
HCC: Hepatocellular carcinoma
HGb: Hemoglobin
IFN-*λ*1/*λ*2: Interferon lambda 1/2
IFN*λ*3: Interferon lambda 3
IQR: Interquartile range
ISGs: Interferon-stimulated genes
LAM: Lamivudine
NAs: Nucleos(t)ide analogues
NEUT: Neutrophils
NSs: Nucleoside analogues
NTs: Nucleotide analogues
PegIFN*α*: PEGylated interferon-alpha
PLT: Platelet
PSM: Propensity score matching
RBC: Red blood cells
SD: Standard deviation
TAF: Tenofovir alafenamide
TBIL: Total bilirubin
TDF: Tenofovir disoproxil fumarate
ULN: Upper limit of normal
WBC: White blood cells.

## Supplementary Materials

Supplementary Materials Table S1: baseline characteristics in the HBeAg-positive subgroup. Table S2: baseline characteristics in the HBeAg-negative subgroup. Table S3: baseline characteristics in the “add-on” subgroup. Table S4: baseline characteristics in the “NAs-experienced” subgroup. Table S5: baseline characteristics of patients with different HBeAg status. Table S6: baseline characteristics of patients with different combination strategies. Table S7: efficacy results at week 48 in the HBeAg-positive subgroup. Table S8: efficacy results at week 48 in the HBeAg-negative subgroup. Table S9: efficacy results at week 48 in the “add-on” subgroup. Table S10: efficacy results at week 48 in the “NAs-experienced” subgroup.
