# Supplementary material for: Reduction of Hepatitis B Surface Antigen May Be More Significant in PEGylated Interferon-Alpha Therapy Combined with Nucleotide Analogues than Combined with Nucleoside Analogues in Chronic Hepatitis B Patients: A Propensity Score Matching Study
Source: Can J Gastroenterol Hepatol. 2022 Dec 7;2022:4325352. doi: 10.1155/2022/4325352 (PMC9750779; doi:10.1155/2022/4325352)
Supplement: Supplementary Materials — Table S1: baseline characteristics in the HBeAg-positive subgroup. Table S2: baseline characteristics in the HBeAg-negative subgroup. Table S3: baseline characteristics in the “add-on” subgroup. Table S4: baseline characteristics in the “NAs-experienced” subgroup. Table S5: baseline characteristics of patients with different HBeAg status. Table S6: baseline characteristics of patients with different combination strategies. Table S7: efficacy results at week 48 in the HBeAg-positive subgroup. Table S8: efficacy results at week 48 in the HBeAg-negative subgroup. Table S9: efficacy results at week 48 in the “add-on” subgroup. Table S10: efficacy results at week 48 in the “NAs-experienced” subgroup. [file 4325352.f1.docx]

TABLE S1: Baseline characteristics in the HBeAg-positive subgroup.

| Variables | PegIFNα + ETV (n = 13) | PegIFNα + NTs (n = 65) | | *P* |
| --- | --- | --- | --- | --- |
|  | PegIFNα + ETV (n = 13) | PegIFNα + ADV (n = 33) | PegIFNα + TDF (n = 32) |  |
| NAs-experienced^c^ | 6 (46.2) | 20 (30.8) | | 0.340 |
|  | 6 (46.2) | 7 (21.2) | 13 (40.6) | 0.134 |
| Weeks of NAs before combined PegIFNα (wk)^b^ | 48 (18–180) | 72 (35–180) | | 0.378 |
|  | 48 (18–180) | 96 (14-456) | 48 (12–72) | 0.373 |
| Weeks of adding-on (wk)^a^ | 12.46 ± 14.75 | 12.03 ± 12.36 | | 0.912 |
|  | 12.46 ± 14.75 | 13.48 ± 10.79 | 10.53 ± 13.81 | 0.645 |
| Total weeks of  combination (wk)^a^ | 35.54 ± 14.75 | 35.97 ± 12.36 | | 0.912 |
|  | 35.54 ± 14.75 | 34.52 ± 10.79 | 37.47 ± 13.81 | 0.111 |
| Age (yr)^a^ | 38.1 ± 6.9 | 33.9 ± 6.8 | | 0.048 |
|  | 38.1 ± 6.9 | 35.0 ± 6.6 | 32.8 ± 6.9 | 0.063 |
| Male^c^ | 12 (92.3) | 48 (73.8) | | 0.278 |
|  | 12 (92.3) | 25 (75.8) | 23 (71.9) | 0.267 |
| BMI (kg/cm^2)^a^ | 23.1 ± 2.0 | 22.4 ± 2.7 | | 0.474 |
|  | 23.1 ± 2.0 | 22.7 ± 2.9 | 22.0 ± 2.5 | 0.469 |
| WBC (x10^9/L)^a^ | 5.6 ± 1.3 | 5.3 ± 1.3 | | 0.474 |
|  | 5.6 ± 1.3 | 5.3 ± 1.6 | 5.3 ± 0.9 | 0.769 |
| NEUT (%)^b^ | 56 (48–63) | 60 (52–67) | | 0.134 |
|  | 56 (48–63) | 54 (48–62) | 57 (49–63) | 0.271 |
| RBC (x10^9/L)^a^ | 5.1 ± 0.8 | 5.0 ± 0.5 | | 0.468 |
|  | 5.1 ± 0.8 | 4.9 ± 0.4 | 5.0 ± 0.6 | 0.486 |
| PLT (x10^9/L)^a^ | 197 ± 54 | 195 ± 42 | | 0.856 |
|  | 197 ± 54 | 184 ± 47 | 205 ± 35 | 0.201 |
| HGb (g/L)^a^ | 157 ± 10 | 152 ± 15 | | 0.271 |
|  | 157 ± 10 | 152 ± 15 | 152 ± 15 | 0.546 |
| ALB (U/L)^a^ | 48 ± 3 | 46 ± 4 | | 0.338 |
|  | 48 ± 3 | 45 ± 3 | 47 ± 4 | 0.130 |
| ALT (U/L)^b^ | 90 (34–203) | 89 (33–217) | | 0.360 |
|  | 90 (34–203) | 170 (71–271) | 90 (36–196) | 0.133 |
| ALT > ULN^c^ | 9 (69.2) | 47 (78.3) | | 0.485 |
|  | 9 (69.2) | 25 (86.2) | 22 (71.0) | 0.277 |
| AST (U/L)^b^ | 45 (24–89) | 39 (23–106) | | 0.410 |
|  | 45 (24–89) | 71 (39–118) | 53 (24–101) | 0.373 |
| GGT (U/L)^b^ | 24 (18–42) | 37 (17–129) | | 0.457 |
|  | 24 (18–42) | 34 (18–62) | 24 (20–36) | 0.321 |
| TBIL (μmol/L)^a^ | 12.3 ± 5.7 | 13.0 ± 7.4 | | 0.737 |
|  | 12.3 ± 5.7 | 11.8 ± 4.5 | 14.2 ± 9.3 | 0.413 |
| HBsAg (log_10_ IU/mL)^a^ | 3.60 ± 0.60 | 3.83 ± 0.72 | | 0.292 |
|  | 3.60 ± 0.60 | 3.88 ± 0.73 | 3.78 ± 0.72 | 0.480 |
| HBsAg＞250 IU/mL^c^ | 13 (100.0) | 63 (96.9) | | 1.000 |
|  | 13 (100.0) | 32 (97.0) | 31 (96.9) | 0.691 |
| HBeAg (s/co)^a^ | 506.22 ± 532.15 | 596.47 ± 628.00 | | 0.630 |
|  | 506.22 ± 532.15 | 731.59 ± 592.90 | 457.13 ± 641.77 |  |
| HBV DNA (log_10_ IU/mL)^a^ | 5.23 ± 2.40 | 5.86 ± 2.09 | | 0.366 |
|  | 5.23 ± 2.40 | 6.41 ± 1.82 | 5.31 ± 2.24 | 0.121 |

a. Variables were expressed as mean ± SD

b. Variables were expressed as median (IQR)

c. Variables were expressed as n (%)

ADV, adefovir dipivoxil; ALB, albumin; ALT, alanine aminotransferase; AST, aspartate transaminase; BMI, body mass index; ETV, entecavir; GGT, gamma glutamyl transferase; HBeAg, hepatitis B e antigen; HBsAg, hepatitis B surface antigen; HGb, hemoglobin; IQR, interquartile range; NAs, nucleos(t)die analogues; NEUT, neutrophils; NSs, nucleoside analogues; NTs, nucleotide analogues; PegIFNα, pegylated interferon α; PLT, platelet; RBC, red blood cells; SD, standard deviation; TBIL, total bilirubin; TDF, tenofovir disoproxil fumarate ; ULN, upper limit of normal; WBC, white blood cells.

TABLE S2: Baseline characteristics in the HBeAg-negative subgroup.

| Variables | PegIFNα + NSs (n = 7) | PegIFNα + NTs (n = 10) | | *P* |
| --- | --- | --- | --- | --- |
|  | PegIFNα + ETV (n = 7) | PegIFNα + ADV (n = 5) | PegIFNα + TDF (n = 5) |  |
| NAs-experienced^c^ | 4 (80.0) | 10 (83.3) | | 1.000 |
|  | 4 (80.0) | 6 (85.7) | 4 (80.0) | 0.954 |
| Weeks of NAs before combined PegIFNα (wk)^b^ | 144 (48–192) | 60 (12–336) | | 0.885 |
|  | 144 (48–192) | 214 (46–348) | 48 (6–264) | 0.427 |
| Weeks of adding-on (wk)^a^ | 4.80 ± 10.73 | 5.00 ± 11.95 | | 0.975 |
|  | 4.80 ± 10.73 | 5.14 ± 13.61 | 4.80 ± 10.7e | 0.998 |
| Total weeks of  combination (wk)^a^ | 39.00 ± 12.37 | 40.00 ± 14.77 | | 0.896 |
|  | 39.00 ± 12.37 | 37.71 ± 17.57 | 43.20 ± 10.73 | 0.687 |
| Age (yr)^a^ | 35.0 ± 4.3 | 39.7 ± 10.4 | | 0.356 |
|  | 35.0 ± 4.3 | 38.0 ± 7.7 | 42.0 ± 14.1 | 0.511 |
| Male^c^ | 5 (100.0) | 11 (91.7) | | 1.000 |
|  | 5 (100.0) | 6 (85.7) | 5 (100.0) | 0.394 |
| BMI (kg/cm^2)^a^ | 24.85 ± 1.80 | 23.44 ± 1.96 | | 0.292 |
|  | 24.85 ± 1.80 | 22.40 ± 0.73 | 25.01 ± 2.28 | 0.046 |
| WBC(x10^9/L)^a^ | 6.2 ± 0.9 | 4.9 ± 1.1 | | 0.030 |
|  | 6.2 ± 0.9 | 4.6 ± 1.1 | 5.2 ± 1.1 | 0.068 |
| NEUT (%)^b^ | 64 (49–71) | 54 (41–68) | | 0.320 |
|  | 64 (49–71) | 54 (46–64) | 53 (38–72) | 0.591 |
| RBC (x10^9/L)^a^ | 5.2 ± 0.5 | 5.0 ± 0.6 | | 0.488 |
|  | 5.2 ± 0.5 | 5.0 ± 1.1 | 5.1 ± 0.5 | 0.709 |
| PLT (x10^9/L)^a^ | 200 ± 18 | 187 ± 42 | | 0.490 |
|  | 200 ± 18 | 188 ± 34 | 185 ± 57 | 0.790 |
| HGb (g/L)^a^ | 155 ± 10 | 154 ± 13 | | 0.810 |
|  | 155 ± 10 | 150 ± 14 | 159 ± 12 | 0.460 |
| ALB (U/L)^a^ | 48 ± 3 | 47 ± 3 | | 0.534 |
|  | 48 ± 3 | 47 ± 3 | 48 ± 3 | 0.830 |
| ALT (U/L)^b^ | 38 (31–49) | 34 (21–86) | | 0.442 |
|  | 38 (31–49) | 23 (20–34) | 40 (28–105) | 0.246 |
| ALT > ULN^c^ | 2 (40.0) | 3 (25.0) | | 0.600 |
|  | 2 (40.0) | 1 (14.3) | 2 (40.0) | 0.498 |
| AST (U/L)^b^ | 24 (22–27) | 23 (20–37) | | 0.959 |
|  | 24 (22–27) | 22 (20–29) | 25 (21–44) | 0.666 |
| GGT (U/L)^b^ | 20 (18–25) | 18 (15–32) | | 0.661 |
|  | 20 (18–25) | 16 (13–18) | 28 (21–35) | 0.047 |
| TBIL (μmol/L)^a^ | 15.6 ± 12.7 | 13.5 ± 4.7 | | 0.774 |
|  | 15.6 ± 12.7 | 14.7 ± 2.6 | 11.6 ± 5.4 | 0.730 |
| HBsAg (log_10_ IU/mL)^a^ | 2.34 ± 1.48 | 2.63 ± 1.17 | | 0.675 |
|  | 2.34 ± 1.48 | 2.67 ± 1.02 | 2.57 ± 1.47 | 0.911 |
| HBsAg＞250 IU/mL^c^ | 2 (40.0) | 8 (66.7) | | 0.593 |
|  | 2 (40.0) | 4 (57.1) | 4 (80.0) | 0.419 |
| HBV DNA (log_10_ IU/mL)^a^ | 3.01 ± 0.62 | 3.31 ± 1.48 | | 0.702 |
|  | 3.01 ± 0.62 | 3.05 ± 0.9 | 3.63 ± 2.08 | 0.729 |

a. Variables were expressed as mean ± SD

b. Variables were expressed as median (IQR)

c. Variables were expressed as n (%)

ADV, adefovir dipivoxil; ALB, albumin; ALT, alanine aminotransferase; AST, aspartate transaminase; BMI, body mass index; ETV, entecavir; GGT, gamma glutamyl transferase; HBeAg, hepatitis B e antigen; HBsAg, hepatitis B surface antigen; HGb, hemoglobin; IQR, interquartile range; NAs, nucleos(t)die analogues; NEUT, neutrophils; NSs, nucleoside analogues; NTs, nucleotide analogues; PegIFNα, pegylated interferon α; PLT, platelet; RBC, red blood cells; SD, standard deviation; TBIL, total bilirubin; TDF, tenofovir disoproxil fumarate ; ULN, upper limit of normal; WBC, white blood cells.

TABLE S3: Baseline characteristics in the "add-on" subgroup.

| Variables | PegIFNα + NSs (n = 8) | PegIFNα + NTs (n = 47) | | *P* |
| --- | --- | --- | --- | --- |
|  | PegIFNα + ETV (n = 8) | PegIFNα + ADV (n = 27) | PegIFNα + TDF (n = 20) |  |
| Weeks of adding-on (wk)^a^ | 23.25 ± 11.21 | 17.91 ± 11.39 | | 0.225 |
|  | 23.25 ± 11.21 | 17.81 ± 9.71 | 18.05 ± 13.61 | 0.105 |
| Total weeks of  combination (wk)^a^ | 24.75 ± 11.21 | 30.09 ± 11.39 | | 0.225 |
|  | 24.75 ± 11.21 | 30.19 ± 9.71 | 29.95 ± 13.61 | 0.482 |
| Age (yr)^a^ | 37.8 ± 6.7 | 33.4 ± 4.7 | | 0.114 |
|  | 37.8 ± 6.7 | 34.0 ± 4.1 | 32.6 ± 5.5 | 0.060 |
| Male^c^ | 7 (87.5) | 34 (72.3) | | 0.664 |
|  | 7 (87.5) | 20 (74.1) | 14 (70.0) | 0.596 |
| BMI (kg/cm^2)^a^ | 22.61 ± 0.81 | 22.53 ± 2.61 | | 0.114 |
|  | 22.61 ± 0.81 | 22.64 ± 2.69 | 22.4 ± 2.60 | 0.953 |
| WBC(x10^9/L)^a^ | 5.8 ± 1.5 | 5.1 ± 1.4 | | 0.219 |
|  | 5.8 ± 1.5 | 5.0 ± 1.7 | 5.3 ± 0.8 | 0.393 |
| NEUT (%)^b^ | 58 (54–65) | 52 (47–60) | | 0.186 |
|  | 58 (54–65) | 52 (45–60) | 55 (49–62) | 0.198 |
| RBC (x10^9/L)^a^ | 4.7 ± 1.0 | 4.9 ± 0.5 | | 0.426 |
|  | 4.7 ± 1.0 | 4.8 ± 0.4 | 5.0 ± 0.6 | 0.473 |
| PLT (x10^9/L)^a^ | 189 ± 64 | 189 ± 43 | | 0.975 |
|  | 189 ± 64 | 180 ± 49 | 202 ± 31 | 0.311 |
| HGb (g/L)^a^ | 155 ± 8 | 152 ± 15 | | 0.447 |
|  | 155 ± 8 | 152 ± 16 | 152 ± 15 | 0.879 |
| ALB (U/L)^a^ | 45 ± 1 | 45 ± 3 | | 0.979 |
|  | 45 ± 1 | 44 ± 3 | 46 ± 4 | 0.346 |
| ALT (U/L)^b^ | 156 (64–257) | 179 (97–267) | | 0.567 |
|  | 156 (64–257) | 206 (85–286) | 152 (97–210) | 0.595 |
| ALT > ULN^c^ | 8 (100.0) | 42 (97.7) | | 1.000 |
|  | 8 (100.0) | 24 (100.0) | 18 (94.7) | 0.366 |
| AST (U/L)^b^ | 104 (36–119) | 77 (49–115) | | 0.848 |
|  | 104 (36–119) | 82 (47–126) | 70 (53–107) | 0.958 |
| GGT (U/L)^b^ | 77 (17–209) | 32 (22–58) | | 0.430 |
|  | 77 (17–209) | 48 (22–66) | 28 (21–33) | 0.323 |
| TBIL (μmol/L)^a^ | 12.1 ± 5.0 | 13.4 ± 8.6 | | 0.698 |
|  | 12.1 ± 5.0 | 11.6 ± 4.8 | 15.9 ± 11.9 | 0.236 |
| HBsAg (log_10_ IU/mL)^a^ | 4.04 ± 0.35 | 4.08 ± 0.59 | | 0.882 |
|  | 4.04 ± 0.35 | 4.11 ± 0.54 | 4.0 ± 0.7 | 0.876 |
| HBeAg (s/co)^a^ | 663.84 ± 508.00 | 736.95 ± 632.54 | | 0.758 |
|  | 663.84 ± 508.00 | 817.72 ± 580.49 | 627.92 ± 696.97 | 0.557 |
| HBV DNA (log_10_ IU/mL)^a^ | 6.65 ± 1.52 | 6.84 ± 1.22 | | 0.706 |
|  | 6.65 ± 1.52 | 7.00 ± 0.96 | 6.7 ± 1.5 | 0.676 |

a. Variables were expressed as mean ± SD

b. Variables were expressed as median (IQR)

c. Variables were expressed as n (%)

ADV, adefovir dipivoxil; ALB, albumin; ALT, alanine aminotransferase; AST, aspartate transaminase; BMI, body mass index; ETV, entecavir; GGT, gamma glutamyl transferase; HBeAg, hepatitis B e antigen; HBsAg, hepatitis B surface antigen; HGb, hemoglobin; IQR, interquartile range; NAs, nucleos(t)die analogues; NEUT, neutrophils; NSs, nucleoside analogues; NTs, nucleotide analogues; PegIFNα, pegylated interferon α; PLT, platelet; RBC, red blood cells; SD, standard deviation; TBIL, total bilirubin; TDF, tenofovir disoproxil fumarate ; ULN, upper limit of normal; WBC, white blood cells.

Table S4: Baseline characteristics in the "NAs- experienced" subgroup.

| Variables | PegIFNα + NSs (n = 10) | PegIFNα + NTs (n = 30) | | *P* |
| --- | --- | --- | --- | --- |
|  | PegIFNα + ETV (n = 10) | PegIFNα + ADV (n = 13) | PegIFNα + TDF (n =17) |  |
| Weeks of NAs before combined PegIFNα (wk)^b^ | 96 (42–168) | 48 (18–240) | | 0.475 |
|  | 96 (42–168) | 96 (24–384) | 48 (14–72) | 0.133 |
| Total weeks of  combination (wk)^a^ | 45.90 ± 6.64 | 46.80 ± 6.57 | | 0.710 |
|  | 45.90 ± 6.64 | 45.23 ± 9.99 | 48.00 ± 0.00 | 0.490 |
| Age (yr)^a^ | 36.8 ± 6.3 | 37.1 ± 10.5 | | 0.933 |
|  | 36.8 ± 6.3 | 38.8 ± 9.8 | 35.8 ± 11.0 | 0.711 |
| Male^c^ | 10 (100.0) | 25 (83.3) | | 0.306 |
|  | 10 (100.0) | 11 (84.6) | 14 (82.4) | 0.209 |
| BMI (kg/cm^2)^a^ | 23.89 ± 2.21 | 22.53 ± 2.64 | | 0.196 |
|  | 23.89 ± 2.21 | 22.68 ± 2.49 | 22.42 ± 2.82 | 0.425 |
| WBC (x10^9/L)^a^ | 5.7 ± 1.0 | 5.4 ± 1.1 | | 0.368 |
|  | 5.7 ± 1.0 | 5.4 ± 1.1 | 5.3 ± 1.1 | 0.650 |
| NEUT (%)^b^ | 65 (51–70) | 61 (53–67) | | 0.516 |
|  | 65 (51–70) | 59 (54–68) | 61 (47–65) | 0.661 |
| RBC (x10^9/L)^a^ | 5.3 ± 0.4 | 5.1 ± 0.6 | | 0.156 |
|  | 5.3 ± 0.4 | 5.0 ± 0.5 | 5.1 ± 0.6 | 0.362 |
| PLT (x10^9/L)^a^ | 206 ± 28 | 199 ± 40 | | 0.645 |
|  | 206 ± 28 | 196 ± 32 | 202 ± 45 | 0.817 |
| HGb (g/L)^a^ | 158 ± 11 | 153 ± 13 | | 0.327 |
|  | 158 ± 11 | 152 ± 11 | 154 ± 15 | 0.595 |
| ALB (U/L)^a^ | 49 ± 3 | 48 ± 3 | | 0.615 |
|  | 49 ± 3 | 48 ± 3 | 48 ± 3 | 0.871 |
| ALT (U/L)^b^ | 36 (26–43) | 30 (22–52) | | 0.646 |
|  | 36 (26–43) | 24 (22–34) | 34 (24–63) | 0.512 |
| ALT > ULN^c^ | 3 (30.0) | 8 (27.6) | | 1.000 |
|  | 3 (30.0) | 2 (16.7) | 6 (35.3) | 0.523 |
| AST (U/L)^b^ | 24 (20–27) | 22 (20–28) | | 0.962 |
|  | 24 (20–27) | 21 (19–28) | 24 (22–28) | 0.444 |
| GGT (U/L)^b^ | 22 (18–33) | 20 (15–33) | | 0.456 |
|  | 22 (18–33) | 16 (12–26) | 23 (16–37) | 0.145 |
| TBIL (μmol/L)^a^ | 13.87 ± 9.45 | 12.75 ± 3.93 | | 0.605 |
|  | 13.87 ± 9.45 | 13.89 ± 3.78 | 11.95 ± 3.94 | 0.585 |
| HBsAg (log_10_ IU/mL)^a^ | 2.62 ± 1.00 | 2.96 ± 0.91 | | 0.322 |
|  | 2.62 ± 1.00 | 2.75 ± 0.81 | 3.12 ± 0.98 | 0.347 |
| HBsAg＞250 IU/mL^c^ | 7 (70.0) | 24 (80.0) | | 0.665 |
|  | 7 (70.0) | 9 (69.2) | 15 (88.2) | 0.355 |
| HBeAg (s/co)^a^ | 158.92 ± 413.28 | 142.71 ± 370.58 | | 0.915 |
|  | 158.92 ± 413.28 | 158.97 ± 383.77 | 129.50 ± 371.63 | 0.974 |
| HBV DNA (log_10_ IU/mL)^a^ | 2.70 ± 0.00 | 3.38 ± 1.65 | | 0.045 |
|  | 2.70 ± 0.00 | 3.74 ± 2.00 | 3.07 ± 1.27 | 0.270 |

a. Variables were expressed as mean ± SD

b. Variables were expressed as median (IQR)

c. Variables were expressed as n (%)

ADV, adefovir dipivoxil; ALB, albumin; ALT, alanine aminotransferase; AST, aspartate transaminase; BMI, body mass index; ETV, entecavir; GGT, gamma glutamyl transferase; HBeAg, hepatitis B e antigen; HBsAg, hepatitis B surface antigen; HGb, hemoglobin; IQR, interquartile range; NAs, nucleos(t)die analogues; NEUT, neutrophils; NSs, nucleoside analogues; NTs, nucleotide analogues; PegIFNα, pegylated interferon α; PLT, platelet; RBC, red blood cells; SD, standard deviation; TBIL, total bilirubin; TDF, tenofovir disoproxil fumarate ; ULN, upper limit of normal; WBC, white blood cells.

Table S5: Baseline characteristics of patients with different HBeAg status.

| Variables | HBeAg-positive (n = 78) | HBeAg-negative (n = 17) | *P* |
| --- | --- | --- | --- |
| NAs-experienced^c^ | 26 (33.3) | 14 (82.4) | 0.001 |
| Weeks of NAs before combined PegIFNα (wk)^b^ | 48 (25–96) | 76 (39–336) | 0.330 |
| Weeks of adding on (wk)^a^ | 12.11 ± 12.68 | 4.9 ± 11.27 | 0.034 |
| Total weeks of  combination (wk)^a^ | 35.90 ± 12.68 | 39.71 ± 13.73 | 0.272 |
| PegIFNα + NTs^c^ | 65 (83.3) | 12 (70.6) | 0.224 |
| PegIFNα + ETV^c^ | 32 (41.0) | 8 (47.1) | 0.648 |
| PegIFNα + ADV^c^ | 14 (17.9) | 4 (23.5) | 0.733 |
| PegIFNα + TDF^c^ | 32 (41.0) | 5 (29.4) | 0.374 |
| Age (yr)^a^ | 34.6 ± 6.9 | 38.3 ± 9.2 | 0.066 |
| Male^c^ | 60 (76.9) | 16 (94.1) | 0.179 |
| BMI (kg/cm^2)^a^ | 22.45 ± 2.61 | 23.77 ± 1.95 | 0.091 |
| WBC(x10^9/L)^a^ | 5.3 ± 1.3 | 5.3 ± 1.2 | 0.812 |
| NEUT (%)^b^ | 56 (50–63) | 56 (44–68) | 0.719 |
| RBC (x10^9/L)^a^ | 5.0 ± 0.5 | 5.1 ± 0.5 | 0.451 |
| PLT (x10^9/L)^a^ | 195 ± 44 | 191 ± 37 | 0.716 |
| HGb (g/L)^a^ | 153 ± 14 | 154 ± 12 | 0.731 |
| ALB (U/L)^a^ | 46 ± 4 | 48 ± 3 | 0.150 |
| ALT (U/L)^b^ | 104 (46–226) | 34 (23–49) | 0.001 |
| ALT > ULN^c^ | 56 (76.7) | 5 (29.4) | 0.001 |
| AST (U/L)^b^ | 53 (26–103) | 24 (20–29) | 0.001 |
| GGT (U/L)^b^ | 28 (20–53) | 20 (16–27) | 0.014 |
| TBIL (μmol/L)^a^ | 12.9 ± 7.1 | 14.1 ± 7.2 | 0.558 |
| HBsAg (log_10_ IU/mL)^a^ | 3.79 ± 0.71 | 2.54 ± 1.22 | 0.001 |
| HBsAg＞250 IU/mL^c^ | 76 (97.4) | 10 (58.8) | 0.001 |
| HBV DNA (log_10_ IU/mL)^a^ | 5.74 ± 2.1 | 3.23 ± 1.3 | 0.001 |

a. Variables were expressed as mean ± SD

b. Variables were expressed as median (IQR)

c. Variables were expressed as n (%)

ADV, adefovir dipivoxil; ALB, albumin; ALT, alanine aminotransferase; AST, aspartate transaminase; BMI, body mass index; ETV, entecavir; GGT, gamma glutamyl transferase; HBeAg, hepatitis B e antigen; HBsAg, hepatitis B surface antigen; HGb, hemoglobin; IQR, interquartile range; NAs, nucleos(t)die analogues; NEUT, neutrophils; NSs, nucleoside analogues; NTs, nucleotide analogues; PegIFNα, pegylated interferon α; PLT, platelet; RBC, red blood cells; SD, standard deviation; TBIL, total bilirubin; TDF, tenofovir disoproxil fumarate ; ULN, upper limit of normal; WBC, white blood cells.

Table S6: Baseline characteristics of patients with different combination strategies.

| Variables | NAs-experienced (n = 40) | add-on (n = 55) | *P* |
| --- | --- | --- | --- |
| HBeAg-positive^c^ | 26 (65.0) | 52 (94.5) | 0.001 |
| PegIFNα + ETV^c^ | 9 (22.5) | 9 (16.4) | 0.597 |
| PegIFNα + ADV^c^ | 15 (37.5) | 25 (45.5) | 0.438 |
| PegIFNα + TDF^c^ | 16 (40.0) | 21 (38.2) | 0.858 |
| PegIFNα + NTs^c^ | 30 (75.0) | 47 (85.5) | 0.199 |
| Total weeks of  combination (wk)^a^ | 46.58 ± 6.52 | 29.31 ± 11.42 | 0.001 |
| Age (yr)^a^ | 37.0 ± 9.5 | 34.0 ± 5.2 | 0.076 |
| Male^c^ | 35 (87.5) | 41 (74.5) | 0.119 |
| BMI (kg/cm^2)^a^ | 22.85 ± 2.58 | 22.54 ± 2.54 | 0.606 |
| WBC (x10^9/L)^a^ | 5.4 ± 1.1 | 5.2 ± 1.4 | 0.388 |
| NEUT (%)^b^ | 61 (51–68) | 54 (48–60) | 0.001 |
| RBC (x10^9/L)^a^ | 5.1 ± 0.5 | 4.9 ± 0.5 | 0.040 |
| PLT (x10^9/L)^a^ | 201 ± 37 | 189 ± 46 | 0.193 |
| HGb (g/L)^a^ | 154 ± 13 | 152 ± 14 | 0.507 |
| ALB (U/L)^a^ | 48 ± 3 | 45 ± 3 | 0.001 |
| ALT (U/L)^b^ | 32 (22–44) | 179 (90–267) | 0.001 |
| ALT > ULN^c^ | 11 (28.2) | 50 (98.0) | 0.001 |
| AST (U/L)^b^ | 23 (20–27) | 79 (48–116) | 0.001 |
| GGT (U/L)^b^ | 20 (15–32) | 33 (21–61) | 0.001 |
| TBIL (μmol/L)^a^ | 13.0 ± 5.6 | 13.2 ± 8.1 | 0.939 |
| HBsAg (log_10_ IU/mL)^a^ | 2.87 ± 0.93 | 4.07 ± 0.56 | 0.001 |
| HBsAg＞250 IU/mL^c^ | 32 (77.5) | 55 (100.0) | 0.001 |
| HBeAg (s/co)^a^ | 146.21 ± 374.26 | 726.32 ± 612.33 | 0.001 |
| HBV DNA (log_10_ IU/mL)^a^ | 3.22 ± 1.46 | 6.81 ± 1.26 | 0.001 |

a. Variables were expressed as mean ± SD

b. Variables were expressed as median (IQR)

c. Variables were expressed as n (%)

ADV, adefovir dipivoxil; ALB, albumin; ALT, alanine aminotransferase; AST, aspartate transaminase; BMI, body mass index; ETV, entecavir; GGT, gamma glutamyl transferase; HBeAg, hepatitis B e antigen; HBsAg, hepatitis B surface antigen; HGb, hemoglobin; IQR, interquartile range; NAs, nucleos(t)die analogues; NEUT, neutrophils; NSs, nucleoside analogues; NTs, nucleotide analogues; PegIFNα, pegylated interferon α; PLT, platelet; RBC, red blood cells; SD, standard deviation; TBIL, total bilirubin; TDF, tenofovir disoproxil fumarate ; ULN, upper limit of normal; WBC, white blood cells.

Table S7: Efficacy Results at Weeks 48 in the HBeAg-positive subgroup.

| Responses | PegIFNα + ETV (n = 13) | PegIFNα + NTs (n = 65) | | *P* （total) | *P* （ETV vs. ADV) | *P* （ETV vs. TDF) | *P* （ADV vs. TDF) |
| --- | --- | --- | --- | --- | --- | --- | --- |
|  | PegIFNα + ETV (n = 13) | PegIFNα + ADV (n = 33) | PegIFNα + TDF (n = 32) |  |  |  |  |
| HBsAg reduction from baseline at wk 48, log_10_ IU/mL | –2.43 | –3.62 | | 0.002 |  |  |  |
|  | –2.43 | –3.65 | –3.59 | 0.008 | 0.003 | 0.005 | 0.811 |
| HBeAg reduction from baseline at wk 48, s/co | –394.33 | –540.56 | | 0.422 |  |  |  |
|  | –394.33 | –654.90 | –422.64 | 0.193 | 0.179 | 0.884 | 0.105 |
| HBV DNA reduction from baseline at wk 48, log_10_ IU/mL | –4.11 | –5.32 | | 0.243 |  |  |  |
|  | –4.11 | –5.99 | –4.62 | 0.158 | 0.093 | 0.646 | 0.127 |
| HBsAg loss, n (%) | 1 (7.7) | 1 (1.5) | | 0.307 |  |  |  |
|  | 1 (7.7) | 0 (0.0) | 1 (3.1) | 0.266 | 0.283 | 0.499 | 0.492 |
| HBeAg loss, n (%) | 3 (23.1) | 11 (16.9) | | 0.693 |  |  |  |
|  | 3 (23.1) | 7 (21.2) | 4 (12.5) | 0.562 | 1.000 | 0.394 | 0.349 |
| HBeAg seroconversion, n (%) | 2 (15.4) | 8 (12.3) | | 0.670 |  |  |  |
|  | 2 (15.4) | 5 (15.2) | 3 (9.4) | 0.742 | 1.000 | 0.617 | 0.708 |
| HBV DNA undetactable, n (%) | 12 (92.3) | 60 (93.8) | | 1.000 |  |  |  |
|  | 12 (92.3) | 28 (87.5) | 32 (100.0) | 0.054 | 1.000 | 0.289 | 0.113 |
| HBsAg reduction > 1 log_10_  from baseline, n (%) | 9 (69.2) | 65 (100.0) | | 0.001 |  |  |  |
|  | 9 (69.2) | 33 (100.0) | 32 (100.0) | 0.001 | 0.004 | 0.005 | constant |
| HBsAg reduction > 1 log_10_  and DNA undetactable, n (%) | 9 (69.2) | 60 (92.3) | | 0.038 |  |  |  |
|  | 9 (69.2) | 28 (84.8) | 32 (100.0) | 0.003 | 0.246 | 0.005 | 0.053 |
| ALT normalization, n (%) | 7 (58.3) | 27 (42.2) | | 0.302 |  |  |  |
|  | 7 (58.3) | 13 (39.4) | 14 (45.2) | 0.527 | 0.258 | 0.438 | 0.641 |

ADV, adefovir dipivoxil; ALT: alanine aminotransferase; ETV: entecavir; HBeAg, hepatitis B e-antigen; HBsAg, hepatitis B surface antigen; HBV, hepatitis B virus; NSs, nucleoside analogues; NTs, nucleotide analogues; PegIFNα, pegylated interferon α; TDF, tenofovir disoproxil fumarate

Table S8: Efficacy Results at Weeks 48 in the HBeAg-negative subgroup.

| Responses | PegIFNα + NSs (n = 5) | PegIFNα + NTs (n = 12) |  | *P* （total) | *P* （ETV vs. ADV) | *P* （ETV vs. TDF) | *P* （ADV vs. TDF) |
| --- | --- | --- | --- | --- | --- | --- | --- |
|  | PegIFNα + ETV (n = 5) | PegIFNα + ADV (n = 7) | PegIFNα + TDF (n = 5) |  |  |  |  |
| HBsAg reduction from baseline at wk 48, log_10_ IU/mL | –2.05 | –2.54 |  | 0.450 |  |  |  |
|  | –2.05 | –2.56 | –2.50 | 0.756 | 0.485 | 0.568 | 0.933 |
| HBV DNA reduction from baseline at wk 48, log_10_ IU/mL | –0.98 | –1.10 | | 0.930 |  |  |  |
|  | –0.98 | –0.80 | –1.47 | 0.902 | 0.913 | 0.772 | 0.663 |
| HBsAg loss, n (%) | 3 (60.0) | 4 (33.3) | | 0.593 |  |  |  |
|  | 3 (60.0) | 2 (28.6) | 2 (40.0) | 0.549 | 0.558 | 1.000 | 1.000 |
| HBV DNA undetactable, n (%) | 5 (100.0) | 12 (100.0) | | constant |  |  |  |
|  | 5 (100.0) | 7 (100.0) | 5 (100.0) |  |  |  |  |
| HBsAg reduction > 1 log_10_  from baseline, n (%) | 4 (80.0) | 11 (91.7) | | 0.515 |  |  |  |
|  | 4 (80.0) | 7 (100.0) | 4 (80.0) | 0.316 | 0.417 | 1.000 | 0.417 |
| HBsAg reduction > 1 log_10_  and DNA undetactable, n (%) | 4 (80.0) | 11 (91.7) | | 0.515 |  |  |  |
|  | 4 (80.0) | 7 (100.0) | 4 (80.0) | 0.316 | 0.417 | 1.000 | 0.417 |
| ALT normalization, n (%) | 2 (40.0) | 6 (50.0) | | 1.000 |  |  |  |
|  | 2 (40.0) | 4 (57.1) | 2 (40.0) | 0.784 | 1.000 | 1.000 | 1.000 |

ADV, adefovir dipivoxil; ALT: alanine aminotransferase; ETV: entecavir; HBeAg, hepatitis B e-antigen; HBsAg, hepatitis B surface antigen; HBV, hepatitis B virus; NSs, nucleoside analogues; NTs, nucleotide analogues; PegIFNα, pegylated interferon α; TDF, tenofovir disoproxil fumarate

Table S9: Efficacy Results at Weeks 48 in the "add-on" subgroup.

| Responses | PegIFNα + ETV (n = 8) | PegIFNα + NTs (n = 47) | | *P* （total) | *P* （ETV vs. ADV) | *P* （ETV vs. TDF) | *P* （ADV vs. TDF) |
| --- | --- | --- | --- | --- | --- | --- | --- |
|  | PegIFNα + ETV (n = 8) | PegIFNα + ADV (n = 27) | PegIFNα + TDF (n =20) |  |  |  |  |
| HBsAg reduction from baseline at wk 48, log_10_ IU/mL | –2.27 | –3.89 | | 0.002 |  |  |  |
|  | –2.27 | –3.91 | –3.85 | 0.008 | 0.003 | 0.005 | 0.863 |
| HBeAg reduction from baseline at wk 48, s/co | –605.72 | –682.18 | | 0.765 |  |  |  |
|  | –605.72 | –757.27 | –584.57 | 0.589 | 0.571 | 0.939 | 0.327 |
| HBV DNA reduction from baseline at wk 48, log_10_ IU/mL | –6.65 | –6.77 | | 0.743 |  |  |  |
|  | –6.65 | –7.00 | –6.50 | 0.600 | 0.588 | 0.816 | 0.324 |
| HBsAg loss, n (%) | 1 (12.5) | 0 (0.0) | | 0.145 |  |  |  |
|  | 1 (12.5) | 0 (0.0) | 0 (0.0) | 0.138 | 0.229 | 0.286 | constant |
| HBeAg loss, n (%) | 2 (28.6) | 6 (13.3) | | 0.291 |  |  |  |
|  | 2 (28.6) | 4 (15.4) | 2 (10.5) | 0.559 | 0.584 | 0.287 | 1.000 |
| HBeAg seroconversion, n (%) | 2 (28.6) | 6 (13.3) | | 0.291 |  |  |  |
|  | 2 (28.6) | 4 (15.4) | 2 (10.5) | 0.559 | 0.584 | 0.286 | 1.000 |
| HBV DNA undetactable, n (%) | 7 (87.5) | 42 (91.3) | | 0.567 |  |  |  |
|  | 7 (87.5) | 22 (84.6) | 20 (100.0) | 0.084 | 1.000 | 0.286 | 0.121 |
| HBsAg reduction > 1 log_10_  from baseline, n (%) | 6 (75.0) | 47 (100.0) | | 0.019 |  |  |  |
|  | 6 (75.0) | 27 (100.0) | 20 (100.0) | 0.017 | 0.047 | 0.074 | constant |
| HBsAg reduction > 1 log_10_  and DNA undetactable, n (%) | 5 (62.5) | 42 (89.4) | | 0.082 |  |  |  |
|  | 5 (62.5) | 22 (81.5) | 20 (100.0) | 0.010 | 0.346 | 0.017 | 0.063 |
| ALT normalization, n (%) | 5 (71.4) | 18 (39.1) | | 0.218 |  |  |  |
|  | 5 (71.4) | 9 (33.3) | 9 (47.4) | 0.173 | 0.097 | 0.391 | 0.337 |

ADV, adefovir dipivoxil; ALT: alanine aminotransferase; ETV: entecavir; HBeAg, hepatitis B e-antigen; HBsAg, hepatitis B surface antigen; HBV, hepatitis B virus; NSs, nucleoside analogues; NTs, nucleotide analogues; PegIFNα, pegylated interferon α; TDF, tenofovir disoproxil fumarate

Table S10: Efficacy Results at Weeks 48 in the " NAs-experienced " subgroup.

| Responses | PegIFNα + NSs (n = 10) | PegIFNα + NTs (n = 30) | | *P* （total) | *P* （ETV vs. ADV) | *P* （ETV vs. TDF) | *P* （ADV vs. TDF) |
| --- | --- | --- | --- | --- | --- | --- | --- |
|  | PegIFNα + ETV (n = 10) | PegIFNα + ADV (n = 13) | PegIFNα + TDF (n = 17) |  |  |  |  |
| HBsAg reduction from baseline at wk 48, log_10_ IU/mL | –2.39 | –2.78 | | 0.264 |  |  |  |
|  | –2.39 | –2.54 | –2.96 | 0.254 | 0.711 | 0.131 | 0.222 |
| HBeAg reduction from baseline at wk 48, s/co | –182.94 | –187.79 | | 0.980 |  |  |  |
|  | –182.94 | –274.66 | –141.02 | 0.793 | 0.696 | 0.840 | 0.500 |
| HBV DNA reduction from baseline at wk 48, log_10_ IU/mL | 0.00 | –1.32 | | 0.021 |  |  |  |
|  | 0.00 | –1.72 | –0.96 | 0.295 | 0.122 | 0.375 | 0.427 |
| HBsAg loss, n (%) | 3 (30.0) | 5 (16.7) | | 0.388 |  |  |  |
|  | 3 (30.0) | 2 (15.4) | 3 (17.6) | 0.928 | 0.618 | 0.638 | 1.000 |
| HBeAg loss, n (%) | 1 (16.7) | 5 (25.0) | | 1.000 |  |  |  |
|  | 1 (16.7) | 3 (42.9) | 2 (15.4) | 0.198 | 0.559 | 1.000 | 0.290 |
| HBeAg seroconversion, n (%) | 0 (0.0) | 2 (10.0) | | 1.000 |  |  |  |
|  | 0 (0.0) | 1 (14.3) | 1 (7.7) | 0.520 | 1.000 | 1.000 | 1.000 |
| HBsAg reduction > 1 log_10_  from baseline, n (%) | 7 (70.0) | 29 (96.7) | | 0.042 |  |  |  |
|  | 7 (70.0) | 13 (100.0) | 16 (94.1) | 0.717 | 0.068 | 0.128 | 1.000 |
| ALT normalization, n (%) | 4 (40.0) | 15 (50.0) | | 0.721 |  |  |  |
|  | 4 (40.0) | 8 (61.5) | 7 (41.2) | 0.466 | 0.414 | 1.000 | 0.269 |

ADV, adefovir dipivoxil; ALT: alanine aminotransferase; ETV: entecavir; HBeAg, hepatitis B e-antigen; HBsAg, hepatitis B surface antigen; HBV, hepatitis B virus; NSs, nucleoside analogues; NTs, nucleotide analogues; PegIFNα, pegylated interferon α; TDF, tenofovir disoproxil fumarate
